# Suplatast Tosilate and Eicosapentaenoic Acid as a Possible Strategy for Maintaining Remission in Minimal Change Nephrotic Syndrome: A Case Report

**DOI:** 10.7759/cureus.48048

**Published:** 2023-10-31

**Authors:** Ayako Y Shichijo, Hirotsugu Iwatani

**Affiliations:** 1 Nephrology, National Hospital Organization Osaka National Hospital, Osaka, JPN

**Keywords:** minimal change nephrotic syndrome, ige, eicosapentaenoic acid, remission, corticosteroid, foxp3, chronic thyroiditis, suplatast tosilate, continuous glucose monitoring, type 1 diabetes

## Abstract

We report a case of a sudden onset of minimal change nephrotic syndrome (MCNS) in a 33-year-old woman with type 1 diabetes mellitus (T1DM), stable microalbuminuria, and chronic thyroiditis. She was successfully treated with intravenous corticosteroids to finally attain a complete remission. Four years later, she also experienced a relapse of MCNS in the same season as the first onset. The chronological levels of serum immunoglobulin E (IgE) showed that extremely high serum IgE levels preceded the onset or the relapse of MCNS, which may suggest an allergic mechanism of MCNS. Eicosapentaenoic acid (EPA) was reported to be beneficial in treating allergic diseases. Suplatast tosilate is an anti-allergic medication that suppresses serum IgE and was reported to be beneficial in reducing corticosteroid dose in nephrotic syndrome in a pilot study. Therefore, during the tapering of corticosteroids to the relapse of MCNS, suplatast tosilate and EPA were administered, and the IgE levels were considerably controlled. The patient was able to maintain remission even after the cessation of corticosteroids. In conclusion, suppressing IgE levels using suplatast tosilate and EPA may be beneficial in maintaining complete remission without corticosteroids in T1DM.

## Introduction

Diabetes mellitus often leads to diabetic nephropathy, but the onset of minimal change nephrotic syndrome (MCNS) in diabetic patients and its relapse is rare. Especially in type 1 diabetes mellitus (T1DM), such complications of MCNS were reported only in a few reports [1]. The relapse of MCNS in T1DM patients is rarely reported [2]. In MCNS, the allergic mechanism is reportedly associated with its development, and high serum immunoglobulin E (IgE) levels at the onset were reported [3]. Moreover, the temporal change of serum IgE levels with regard to the onset or relapse is rarely reported.

Here, we report a case of both an onset and a relapse of MCNS in a patient with T1DM. She was successfully treated with intravenous corticosteroid therapy to finally attain complete remission. Continuous glucose monitoring (CGM) was performed, and the chronological change of IgE levels was also monitored.

Eicosapentaenoic acid (EPA) was reported to be beneficial in treating allergic diseases [4,5]. Suplatast tosilate is an anti-allergic medication that suppresses IgE and was reported to be beneficial in tapering corticosteroid doses in nephrotic syndrome in a pilot study [6]. Therefore, we administered suplatast tosilate and EPA during the tapering phase of corticosteroids for the relapse of MCNS, which seemed to be triggered by allergic stimuli, and investigated whether suppressing serum IgE levels using suplatast tosilate and EPA can be an alternative to corticosteroids in maintaining remission of MCNS in T1DM.

## Case presentation

A 33-year-old female had been treated for T1DM with insulin for 16 years. Her urinary albumin excretion had been ≤10 mg/gCr but increased to 89.7 mg/gCr in January 2015. Although it gradually decreased to 21.6 mg/gCr over two months, it suddenly increased to 2140 mg/gCr in August 2015, and she was referred to the Department of Nephrology.

At the first visit to our department, she also presented with edema. We also confirmed that she was also diagnosed with chronic thyroiditis recently. Her family history showed that her mother had Graves' disease and her sister had T1DM. However, no goiter and thyroid hormones were within the normal range. Low serum albumin level (3.0 g/dl) and hyperlipidemia (low-density lipoprotein cholesterol 187 mg/dl) were confirmed. She was suspected of nephrotic syndrome. Diabetic retinopathy in both fundi was mild (simple diabetic retinopathy). Diabetic neuropathy was unlikely because the coefficient of variation of the R-R interval at rest on the electrocardiogram was 10.1%. She complained of a cough in April 2015, and allergic rhinitis was also diagnosed. Her serum IgE level was 1080 mg/dl, but no sinusitis was observed. Her lower right buried tooth was extracted to eradicate the possible focal infection, but urinary albumin excretion did not decrease in 69 days. The selectivity index was 0.06, classified as high urinary albumin selectivity, and we performed a renal biopsy 35 days after the first visit. Laboratory data in detail are shown in Table 1.

**Table 1:** Laboratory data at renal biopsy and the relapse BUN: blood urea nitrogen, Cr: creatinine, T-Chol: total cholesterol, LDL-C: low-density lipoprotein cholesterol, HbA1c: hemoglobin A1c, IgG: immunoglobulin G, IgM: immunoglobulin M, IgA: immunoglobulin A, IgE: immunoglobulin E, TSH: thyroid-stimulating hormone, CRP: C-reactive protein, WBC: white blood cell, RBC: red blood cell, Hb: hemoglobin, Plt: platelet, ESR: erythrocyte sedimentation rate, ND: not done

	Reference range	At a renal biopsy in September 2015	At the relapse in April 2019
Total protein (g/dL)	6.6-8.1	6.1	5.1
Albumin (g/dL)	4.1-5.1	3.0	2.2
BUN (mg/dL)	8-20	12	13
Cr (mg/dL)	0.65-1.07	0.58	0.77
T-Chol (mg/dL)	142-248	289	318
LDL-C (mg/dL)	65-163	187	209
Glucose (mg/dL)	73-109	153	245
HbA1c (%)	4.9-6.0	8.2	8.3
IgG (mg/dL)	861-1747	1274	852
IgM (mg/dL)	50-269	184	139
IgA (mg/dL)	93-393	298	201
IgE (mg/dL)	0-173	1080	812
C3 (mg/dL)	73-138	133	107
C4 (mg/dL)	11-31	33	26
CH50 (CH50/mL)	25.0-48.0	44.1	41.1
F - T3 (pg/mL)	2.30-4.00	2.67	2.43
F - T4 (ng/dL)	1.10-1.80	1.20	1.03
TSH (μIU/mL)	0.27-4.20	0.98	1.99
CRP (mg/dL)	0.00-0.14	0.14	0.07
WBC (/μL)	3300-8600	3300	4100
RBC (10^4^/μL)	435-555	432	453
Hb (g/dL)	13.7-16.8	12.5	14.8
Plt (10^4^/μL)	15.8-34.8	18.3	19.8
ESR 1h/2h (mm)	3-15 mm/h	N.D.	28/56
Urinary protein (dip stick)	(-)	(3+)	(4+)
Urinary blood (dip stick)	(-)	( - )	(+)
Urinary glucose (dip stick)	(-)	(4+)	(+)
Urinary protein (g/gCr)	<0.15	2.496	13.16
Urinary albumin (mg/gCr)	<30	1875.6	N.D.
Selectivity index	-	0.08	0.139

**Figure 1:**
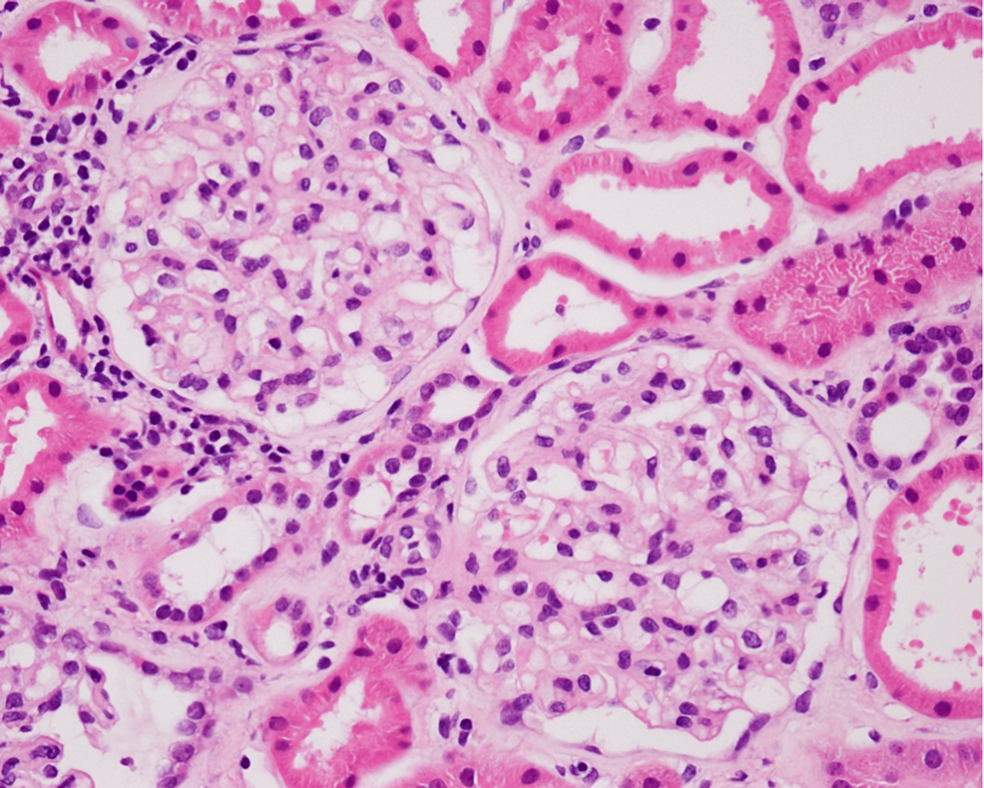
Renal pathology Light micrograph of glomeruli shows minor glomerular abnormalities (H&E stain, x200)

The renal pathology revealed minor glomerular abnormalities (Figure 1).

**Figure 2:**
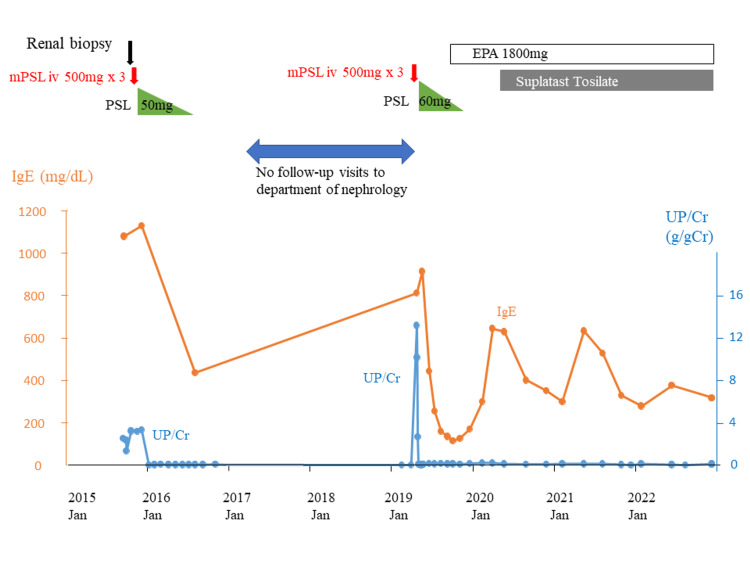
Clinical course mPSL: methylprednisolone, PSL: prednisolone, EPA: eicosapentaenoic acid, Jan: January, UP/Cr: urinary protein

Five hundred milligrams of methylprednisolone (mPSL) were intravenously administered for three days, followed by 50 mg/day of oral prednisolone (PSL). The urinary protein disappeared seven days after the start of corticosteroids, and PSL was gradually tapered. She had been in stable remission, leading to the discontinuation of PSL seven months after the start of the intravenous administration. Blood glucose levels were continuously monitored using a sensor-augmented pump (SAP) during admission, which showed elevated glucose levels during steroid administration, although blood glucose was controlled by SAP under the supervision of the department of diabetes mellitus. The clinical course of nephrotic syndrome is illustrated in Figure 2.

In February 2019, almost four years after the first onset of nephrotic syndrome, she complained of dry cough and symptoms of allergic rhinitis. Two months later, she presented with leg edema. She gained 7 kg of body weight, and urinary protein increased remarkably to 13 g/gCr. Her selectivity index was 0.139, which was categorized as high selectivity again. She was then diagnosed as a relapse of MCNS and was intravenously administered 500 mg of mPSL for three days, followed by 60 mg/day of intravenous PSL. Her urinary albumin decreased promptly again in five days, and PSL was gradually tapered.

Seven months after administering 500 mg of mPSL to this relapse, corticosteroids were discontinued. Subsequently, urine protein remained around 0.2 g/gCr, and she has been in complete remission for 43 months.

## Discussion

We encountered a sudden onset of nephrotic syndrome in one patient who had T1DM with stable microalbuminuria and a past history of chronic thyroiditis. This clinical course was not indicative of the natural course of the typical diabetic nephropathy, manifested as the gradual increase of edema or proteinuria. We had to suspect the complication or the superimposed form of primary glomerulonephritis and needed to perform a renal biopsy. The renal histology was minor glomerular abnormalities, highly suggestive of MCNS.

The histological characteristics were further strengthened by the highly albumin-selective proteinuria indicated by a low selectivity index value. As expected by the renal histology and highly selective proteinuria, the response to corticosteroid therapy was prompt and excellent, leading to a complete remission within seven days after the start of the corticosteroid therapy. When our patient was treated with corticosteroid pulse therapy and the subsequent oral PSL for MCNS, her blood glucose level was continuously monitored using SAP. Although blood glucose monitoring using a CGM device in T1DM patients was reported [7], CGM in T1DM patients with nephrotic syndrome undergoing corticosteroid pulse is rare, and this is the first report to the best of our knowledge. The basal glycemic control had been poor, as shown by HbA1c of around 8%. The corticosteroid pulse therapy and the subsequent massive corticosteroid therapy induced elevated blood glucose, especially on the second day of the corticosteroid pulse therapy. Hypoglycemia was sometimes observed.

We also experienced a recurrence of MCNS in the same patient four years later in the same season. In our case, her serum IgE level rose when she developed MCNS for the first time, decreased as MCNS was in remission, and rose again at the time of recurrence (Figure 2). The development or relapse of MCNS can be traced back to January or February, with preceding symptoms of allergic rhinitis, and the association between the allergic mechanism and the development of MCNS was considered as was reported previously [2]. The abnormal elevation of IgE and the association of the allergic mechanism may underline the onset of MCNS [3]. Nishizono et al. also reported high serum IgE levels at the occurrence and relapse of MCNS [2]. The strong association of allergic mechanism, as was revealed by the preceding rise in serum IgE at the phase of the onset and relapse of MCNS and the fall in IgE at the phase of remission in our case, inspired us to suppress the allergic activity to prevent the future possible relapse of MCNS, especially when corticosteroids are off.

Suplatast tosilate is known to suppress IgE production, the invasion of eosinophils, and the synthesis of IL-4 and IL-5, leading to the suppression of allergic activities, which can be used to treat asthma, allergic rhinitis, or atopic dermatitis. Moreover, its ability to prevent the relapse of idiopathic nephrotic syndrome and its impact on administering corticosteroids at lower doses have already been reported [6]. We administered suplatast tosilate to treat her allergic rhinitis, which she had after the remission to the relapse to suppress allergic activity, hoping this may prevent further relapse of MCNS.

We also administered EPA to treat her hypertriglyceridemia, which she had after the remission to the relapse. EPA is one of the n-3 fatty acids richly contained in fish oil. Fish oil is reported to have beneficial effects on lipids and renal disease in nephrotic rats [8]. The low frequency of asthma in Eskimos may be due to the large intake of fish or seals that contain n-3 fatty acids [9]. A beneficial effect of n-3 fatty acids in inﬂammatory diseases, specifically asthma, has recently been reported [4]. Moreover, the oral administration of EPA reportedly improved asthma symptom scores and significantly suppressed IL-17A and TNF-alpha levels [10]. In this sense, n-3 fatty acids are potentially beneficial to managing asthma, an allergic and inflammatory disease [5].

The temporal change of both serum IgE levels and the amount of proteinuria for more than several years in a patient with MCNS provides us with many important suggestions to understand the mechanism of the disease and the future possible treatment options. This idea of perceiving MCNS from the temporal change of serum IgE and proteinuria is a novel and vital point in our case report. In 2020, the serum IgE levels did not surpass 800 mg/dL, which was the level at the relapse of MCNS in 2019. This may be the prolonged effect of the corticosteroid therapy ceased in 2019. EPA was started during the tapering of corticosteroids in 2019, and suplatast tosilate was further added to EPA just around the peak season of IgE in 2020, which might have led to the further great suppression of the serum IgE at the peak season in 2022. We hope that the concomitant use of suplatast tosilate and EPA without corticosteroids may be beneficial in preventing relapse of nephrotic syndrome and maintaining remission for more than three years despite the periodical slight increase of serum IgE levels every spring.

Regarding the background of our present case, T1DM and chronic thyroiditis are known as autoimmune disorders, and the concomitant occurrence of MCNS was reported [1,11]. Our patient also has a family history of T1DM and thyroid disease. Reportedly, a reduced thyroid reserve may predispose patients to hypothyroidism in nephrotic syndrome [12]. Furthermore, the regulatory T cell (Treg cell) dysfunction is suggested to be crucial for the development of MCNS [13]. In the report, a five-year-old boy with IPEX syndrome with mutations in the FOXP3 gene had manifestations such as atopic dermatitis, T1DM, enteropathy, sepsis, and hemolytic anemia. He was complicated by MCNS. This case of IPEX syndrome has many manifestations in common with our present case, such as allergic disorders, T1DM, high IgE levels, and MCNS. Our present case suggests that suppressing IgE levels with suplatast tosilate and EPA can be important for avoiding the relapse of MCNS without using corticosteroids. This is reasonable considering that the Treg cell dysfunction might be under the pathogenesis for developing this disease condition. However, the mutations in the FOXP3 gene are not investigated in our case.

We previously reported a case of MCNS, which was completely resolved by controlling chronic sinusitis without corticosteroids, which suggested the importance of controlling focal infection [14]. In the present case, the lower right buried tooth was extracted to eradicate the possible focal infection that might have affected nephrotic syndrome, but urinary protein excretion did not decrease. In our case, the lower right buried tooth was not a focus of focal infection.

## Conclusions

We experienced sudden onset and relapse of MCNS in an adult patient with T1DM and chronic thyroiditis, managed successfully by corticosteroid pulse therapy. The temporal change of serum IgE levels, which sharply increased at the onset and the relapse, suggested an allergic mechanism of MCNS. Suppressing IgE levels with suplatast tosilate and EPA might be an alternative to corticosteroids in maintaining complete remission of MCNS. In the near future, however, further research is required to prove this.
